# Early Experience Using Proton Beam Therapy for Extremity Soft Tissue Sarcoma: A Multicenter Study

**DOI:** 10.14338/IJPT-21-00037.1

**Published:** 2022-05-06

**Authors:** Brady S. Laughlin, Michael A. Golafshar, Safia Ahmed, Matthew Prince, Justin D. Anderson, Tamara Vern-Gross, Mahesh Seetharam, Krista Goulding, Ivy Petersen, Todd DeWees, Jonathan B. Ashman

**Affiliations:** 1 Department of Radiation Oncology, Mayo Clinic, Phoenix, AZ, USA; 2 Department of Radiation Oncology, Mayo Clinic, Rochester, MN, USA; 3 Department of Hematology/Oncology, Mayo Clinic, Phoenix, AZ, USA; 4 Department of Orthopedic Surgery, Mayo Clinic, Phoenix, AZ, USA

**Keywords:** protons, extremity soft tissue sarcoma, acute wound complication

## Abstract

**Purpose:**

Proton beam therapy (PBT) may provide an advantage when planning well-selected patients with extremity soft tissue sarcoma (eSTS), specifically for large, anatomically challenging cases. We analyzed our early experience with PBT on toxicity and outcomes.

**Materials and Methods:**

A retrospective study was performed for eSTS treated between June 2016 and October 2020 with pencil beam scanning PBT at 2 institutions. Diagnostic, treatment, and toxicity characteristics were gathered from baseline to last follow-up or death. Wound complications were defined as secondary operations for wound repair (debridement, drainage, skin graft, and muscle flap) or nonoperative management requiring hospitalization. Statistical analysis was performed with R software.

**Results:**

Twenty consecutive patients with a median age 51.5 years (range, 19–78 years) were included. Median follow-up was 13.7 months (range, 1.7–48.1 months). Tumor presentation was primary (n = 17) or recurrent after prior combined modality therapy (n = 3). Tumor location was either lower extremity (n = 16) or upper extremity (n = 4). Radiation was delivered preoperatively in most patients (n = 18). Median pretreatment tumor size was 7.9 cm (range, 1.3 –30.0 cm). The 1-year locoregional control was 100%. Four patients (20%) had developed metastatic disease by end of follow-up. Maximum toxicity for acute dermatitis was grade 2 in 8 patients (40%) and grade 3 in 3 patients (15%). After preoperative radiation and surgical resection, acute wound complications occurred in 6 patients (35%). Tumor size was larger in patients with acute wound complications compared with those without (medians 16 cm, range [12–30.0 cm] vs 6.3 cm, [1.3–14.4 cm], *P* = .003).

**Conclusion:**

PBT for well selected eSTS cases demonstrated excellent local control and similar acute wound complication rate comparable to historic controls. Long-term follow-up and further dosimetric analyses will provide further insight into potential advantages of PBT in this patient population.

## Introduction

Limb sparing surgery remains the primary modality for treatment of extremity soft tissue sarcoma (eSTS) [1, 2]. Despite improvements in imaging and surgical technique, radiation continues to be an integral part of the treatment paradigm to improve local control [3]. However, radiotherapy (RT) can be associated with both short-term and long-term treatment related morbidity that ultimately impacts a patient's function and quality of life [4–6]. In the past decade, outcomes have been improved through advances in RT planning and delivery techniques. Intensity modulated RT (IMRT) has shown the potential to reduce toxicities and improve tumor control compared to conventionally planned RT [7–9]. Image-guided RT combined with smaller target margins demonstrated reduced grade ≥ 2 lymphedema, fibrosis, and joint stiffness in a phase II trial [9].

Proton beam therapy (PBT) is another potential option to further improve the conformality of radiation dose to help minimize treatment morbidity. PBT could be advantageous in certain patients with eSTS given the improved target conformality and reduction in integral dose [3, 10, 11]. The possible benefits of PBT compared with photons for patients with eSTS would be the potential reduction in dose to bone while sparing lymphatic channels and limiting dose to the circumferential limb. While an advantage of PBT has been demonstrated for multiple types of sarcomas including chordoma, chondrosarcoma, osteosarcoma, and Ewing sarcoma, a specific focus on its role in the treatment of eSTS is lacking [12–14]. In this study, we review our initial multicenter experience using PBT in the setting of newly diagnosed or recurrent eSTS.

## Materials and Methods

Institutional review board approval was obtained before performing this retrospective analysis. Patients treated at 2 proton centers with preoperative or postoperative PBT for eSTS were analyzed from June 27, 2016, to October 12, 2020. A gross total resection was performed in all patients. Chemotherapy was prescribed neoadjuvantly, concurrently, or adjuvantly at the discretion of the medical oncologist. Intraoperative radiation boost was delivered when it was felt to be beneficial by the surgeon and radiation oncologist.

### Radiation Treatment Planning and Delivery

Patients were simulated with computed tomography in treatment position. Optimal positioning for stable and reproducible setup was determined based tumor location and size. Immobilization was achieved using a custom fabricated cradle and thermoplastic mask as appropriate for each individual patient's anatomy. Immobilization setup was evaluated for reproducibility by a physician, proton physicist, and proton dosimetrist. Contralateral extremities, particularly lower extremities, were left in the natural position. Intravenous contrast was used unless contraindicated. Volume and organs at risk definition and treatment planning was performed in Eclipse (Varian Medical Systems, Crawley, UK). Gross tumor volume was delineated based on the computed tomography simulation and image fusion with magnetic resonance imaging and positron emission tomography imaging when available. The gross tumor volume was expanded to create the clinical target volume (CTV) according to consensus guidelines to include adjacent tissue at risk for microscopic extension [15]. The CTV was constrained by anatomic barriers, including fascia, bone, or compartment. The circumferential soft tissue and adjacent bone were contoured as organs at risk. Beam specific optimization target volumes and scanning target volumes were created during the planning process.

The proton plans were generated using pencil beam spot scanning using single-field or multifield optimization [16, 17]. Typical field arrangements used 2 to 3 fields, all treated daily with consideration of beam angles to optimize normal tissue sparing and to minimize potential high biologic dose at the distal end of each beam. Target coverage was evaluated to the CTV with 3 mm/3% robustness scenarios to account for daily setup variability and beam range uncertainty. Dose prescription was described in centigray radiobiologic equivalent (cGy RBE). Seventeen available proton plans were evaluated by using dose-volume histogram data for target volume and organs at risk. The dose volume parameters evaluated include mean dose to CTV, bone, and soft tissue.

### Endpoints

The primary outcome measured for this study was acute complication rate. Acute wound complications were defined as a secondary operation for wound repair, such as debridement, skin graft, or muscle flap or nonoperative management, such as the use of intravenous antibiotics requiring hospitalization occurring within 3 months of wide local excision [4]. Skin toxicity during radiation treatment, and long-term pain, peripheral sensory neuropathy, fibrosis, lymphedema, and fracture were evaluated per the Common Terminology Criteria for Adverse Events version 5.0 [18]. Length of follow-up was determined as the time from completion of all treatment to date of last follow-up. Treatment effect was defined as a substantial decrease in viable tumor seen at final pathology after neoadjuvant treatment.

Statistical analysis was performed using R software version 4.0.3 (R Foundation for Statistical Computing, Vienna, Austria) [19]. Descriptive statistics were generated to determine baseline characteristics pertaining to diagnosis, treatment, outcomes, and toxicity. Tests for group differences by acute wound complication were conducted using the Fisher exact test for differences in rates of categorical variables and Kruskal-Wallis rank sum tests to compare distributions of continuous variables.

## Results

### Patient Characteristics

All patients with eSTS in this study were treated with pencil beam scanning PBT (n=20; **Table 1**). The median age at start of treatment was 51.5 years old (range, 19–78 years). Thirteen (65%) patients had sarcoma of upper leg, 3 (15%) patients had sarcoma of lower leg, 3 (15%) patients had sarcoma of upper arm, and 1 (5%) patient had sarcoma of lower arm. Of patients with a proximal lower extremity sarcoma, 9 (69%) were in the upper medial thigh/groin. Seventeen patients were treated for primary tumor, and 3 patients were treated for recurrent tumor. Before radiation, median size on imaging was 9.2 cm (1.3–30.0 cm). Stage distribution was as follows: 2 (10%) IA, 5 (25%) IB, 5 (25%) II, 4 (20%) IIIA, and 4 (20%) IIIB. The diagnosis was made by a biopsy specimen in 14 (70%) patients and after unplanned excision in 6 (30%) patients.

**Table 1.** Patient and tumor characteristics.

**Characteristic**	**Overall, n (%) (N = 20)**
Location of mass	
Upper leg^a^	13 (65.0)
Lower leg	3 (15.0)
Upper arm	3 (15.0)
Lower arm	1 (5.0)
Location on limb	
Distal	3 (15.0)
Proximal	17 (85.0)
Presentation	
Primary	17 (85.0)
Recurrence	3 (15.0)
Tumor size (cm)^b^	
Mean (SD)	9.8 (7.8)
Median	7.4
Range	1.3–30.0
Histology type	
Leiomyosarcoma	1 (5.0)
Malignant peripheral nerve sheath tumor	2 (10.0)
Myxofibrosarcoma	3 (15.0)
Myxoid liposarcoma	3 (15.0)
Other	2 (10.0)
Synovial sarcoma	3 (15.0)
Undifferentiated pleomorphic Sarcoma	2 (10.0)
Well-differentiated liposarcoma	4 (20.0)
Wound closure procedure/type	
Flap	12 (63.1)
Primary	7 (36.9)
Missing	1
Surgical margin status^c^	
R0	19 (68.4)
Clinical T stage	
T1	7 (35.0)
T2	5 (25.0)
T3	5 (25.0)
T4	3 (15.0)
Clinical N stage	
N0	19 (95.0)
Not reported	1 (5.0)
Clinical M Stage	
M0	20 (100.0)
Grade	
Grade 1 (well differentiated)	7 (35.0)
Grade 2 (moderately differentiated)	2 (10.0)
Grade 3 (poorly differentiated)	11 (55.0)
Overall clinical stage [20]	
IA	2 (10.0)
IB	5 (25.0)
II	5 (25.0)
IIIA	4 (20.0)
IIIB	4 (20.0)
Initial unplanned excision	
Yes	6 (30.0)
No	14 (70.0)

a Nine patients had tumor located in upper medial thigh or groin.

b Largest diameter measured on initial imaging.

c One preoperative patient did not go on to surgery.

Neoadjuvant chemotherapy was delivered for 5 (25%) patients. Of patients, 1 received rituximab, cyclophosphamide, doxorubicin hydrochloride, vincristine sulfate, and prednisone chemotherapy for a concurrently diagnosed lymphoma. Neoadjuvant regimens for eSTS were doxorubicin and ifosfamide (n = 2), doxorubicin and dacarbazine (n = 1), and doxorubicin and epirubicin (n = 1). Concurrent chemotherapy was provided for 4 (20%) patients. Rituxan was given as concurrent chemotherapy to 1 (5%) patient. The other 3 patients received concurrent mitomycin, doxorubicin, and cisplatin chemotherapy. Mitomycin, doxorubicin, and cisplatin chemotherapy was continued adjuvantly for 1 patient (5%).

Eighteen (90%) patients received PBT preoperatively and 2 (10%) were treated postoperatively (**Table 2**). Of patients diagnosed by unplanned excision, radiation was delivered before (n = 4) or after (n = 2) formal re-excision. The median proton beam dose was 5000 cGy RBE in 25 fractions (range, 3000–6600 cGy RBE). One patient did receive 4 fractions of photon radiation before switching to PBT. Of 2 patients receiving postoperative PBT, 1 received 6000 cGy RBE and the other received 6600 cGy RBE. Three (15%) patients received PBT as reirradiation in the setting of recurrent disease after prior external beam radiation and surgery at the time of initial diagnosis. One patient received 3000 cGy RBE 1 year after receiving the first course of RT. Two patients received 5000 cGy RBE reirradiation after a period of 2 and 6 years after the first course of radiation. Intraoperative electron RT was delivered to the surgical bed in 9 patients. The median intraoperative electron RT dose was 1250 cGy (range, 950–1750 cGy). Median electron energy was 9 MeV (range, 6–9 MeV) and prescribed to the 90% isodose line.

**Table 2.** Treatment characteristics for patients with extremity STS treated with protons.

**Parameter**	**Overall (N = 20)**
Timing of radiation, n (%)	
Postoperative	2 (10.0)
Preoperative	18 (90.0)
Reirradiation, n (%)	
Yes	3 (15.0)
No	17 (85.0)
Previous treatment, n (%)	
Photons	3 (100.0)
Previous dose, n (%)	
5000	2 (66.7)
6000	1 (33.3)
Age at RT start	
Mean (SD)	49.4 (19.9)
Median	51.5
Range	19.0–78.0
Total proton dose, n (%), cGy	
3000	1 (5.0)
4500	1 (5.0)
5000	16 (80.0)
6000	1 (5.0)
6600	1 (5.0)
IORT	9 (45)
IORT dose	
Median	1250.0
Range	950.0–1750.0
Electron energy, n (%), MeV	
6	2 (22.2)
9	7 (77.8)

**Abbreviations:** STS, soft tissue sarcoma; RT, radiotherapy; cGy, centigray; IORT, intraoperative electron radiotherapy.

One (5%) patient did not undergo surgery after completing radiation due to the development of metastatic disease and was subsequently started on systemic therapy. In patients receiving neoadjuvant radiation, the median time from PBT to surgery was 1.2 months (range, 0.8–1.5 months). The range from surgery to PBT start in the 2 patients receiving adjuvant radiation was 2.1 to 6.9 months. Of 19 patients that underwent surgical resection, primary closure was performed in 7 patients (36.9%) and flap for wound closure was performed in 12 of 19 patients (63.1%). Final surgical margins were negative as defined by no tumor on ink in all 19 (100%) patients. Of the 19 surgical patients, 6 (31.5%) patients had close margins with tumor less than 2 mm from ink.

### Outcomes

The median follow-up was 13.7 months (range, 1.7–48.1 months). In patients who did not undergo any surgical intervention before RT, treatment effect was appreciated in 10 of 13 (77%) patients. Of 20 patients, 1 patient died 11.5 months after radiation alone due to metastatic disease. During follow-up, 4 of 19 patients had developed metastatic disease. No patient experienced a local failure.

### Toxicity

During PBT, grade 2 and 3 acute dermatitis occurred in 8 (40%) and 3 (15%) patients, respectively. Rates of chronic G2+ pain, peripheral sensory neuropathy, fibrosis, and lymphedema were 26%, 16%, 5%, and 5%, respectively. Rates of chronic G3+ pain, peripheral sensory neuropathy, fibrosis, and lymphedema were 0%, 6%, 6%, and 0%, respectively. One patient received an initial course of postoperative PBT to 66 Gy to the hip and then required palliative photon radiation (20 Gy in 5 fractions) to the leg for a metastatic lesion 6 months later; subsequently, this patient developed a pathologic fracture 6 months after second course of RT. There were no grade 4 to 5 acute or late toxicities.

**Figure 1.**
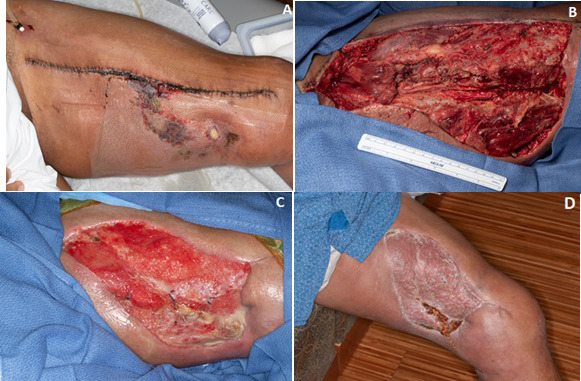
This patient developed severe skin necrosis requiring skin grafting.

Acute wound complications occurred in 6 of 17 patients undergoing preoperative PBT followed by surgical excision (35%). All patients had tumors located in the lower extremity as follows: 5 occurring in proximal lower extremity (thigh) and 1 distal lower extremity (foot) (**Figure 1**; **Table 3**). Intraoperative debridement and incision and drainage, unplanned skin graft, muscle flap, and intravenous antibiotic use requiring hospitalization were required in 6 (30%), 2 (10%), 2 (10%), and 2 (10%) patients, respectively. Larger tumor size was significantly associated with acute wound complications compared with those without (median 16 cm [range, 12.0–30.0 cm] vs 6.3 cm [1.3–14.4 cm], *P* = .003). Low-grade tumors also were associated with wound complications because these patients were more likely to present with larger tumors. Patient age, tumor location, histology, chemotherapy, or intraoperative electron RT were not associated with wound complications. There were no wound complications in the 2 patients who received postoperative PBT.

**Table 3.** Analysis of factors associated with acute wound complications after preoperative PBT.

**Parameter**	**Yes (N = 6)**	**No (N = 11)**	**Total (N = 17)**	***P*** **value**
Tumor location, n (%)				.515^1^
Lower extremity	6 (100.0)	8 (72.7)	14 (82.4)	
Upper extremity	0 (0.0)	3 (27.3)	3 (17.6)	
Tumor location, n (%)				>.99^a^
Distal	1 (16.7)	2 (18.2)	3 (17.6)	
Proximal	5 (83.3)	9 (81.8)	14 (82.4)	
Tumor size^c^ size on imaging (largest dimension in cm)				.003^b^
Mean (SD)	19.5 (7.6)	6.1 (3.9)	10.3 (8.2)	
Median	16.0	6.3	7.9	
Range	12.0–30.0	1.3–14.4	1.3–30.0	
Clinical T-stage [20], n (%)				.031^a^
T1	1 (16.7)	5 (45.5)	6 (35.3)	
T2	0 (0.0)	4 (36.4)	4 (23.5)	
T3	2 (33.3)	2 (18.2)	4 (23.5)	
T4	3 (50.0)	0 (0.0)	3 (17.6)	
Grade				.035^a^
Grade 1 (well differentiated)	5 (83.3)	2 (18.2)	7 (41.2)	
Grade 2 (moderately differentiated)	0 (0.0)	1 (9.1)	1 (5.9)	
Grade 3 (poorly differentiated)	1 (16.7)	8 (72.7)	9 (52.9)	
Overall clinical stage [20], n (%)				.074^a^
IA	1 (16.7)	1 (9.1)	2 (11.8)	
IB	4 (66.7)	1 (9.1)	5 (29.4)	
II	0 (0.0)	4 (36.4)	4 (23.5)	
IIIA	0 (0.0)	3 (27.3)	3 (17.6)	
IIIB	1 (16.7)	2 (18.2)	3 (17.6)	

**Abbreviation:** PBT, proton beam therapy.

a Fisher exact test for count data.

b Kruskal-Wallis rank sum test.

c Largest diameter measured on initial imaging.

### Dosimetry

**Figure 2.**
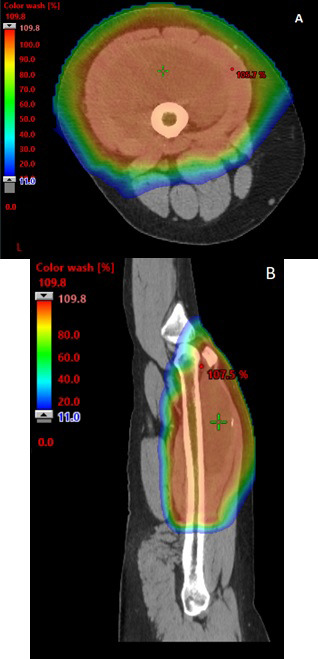
Axial (A) and sagittal (B) views of proton dose colorwash for extremity sarcoma of left thigh.

Dose volume histogram data were analyzed for the 17 patients treated with preoperative PBT and surgery. Target coverage was met for all cases. The mean dose to the nontarget circumferential soft tissue was 2.1 Gy and the mean dose to the adjacent bone was 14.1 Gy. The ability to limit circumferential dose with PBT is demonstrated in **Figure 2** for a representative patient with a large myxoid liposarcoma.

## Discussion

To our knowledge, this is the first series to analyze pencil beam scanning PBT specifically as part of combined modality treatment of eSTS. Our results suggest that pencil beam scanning PBT is a potentially safe and effective option for appropriately selected patients with eSTS. Prior publications have included a limited number of pencil beam scanning PBT cases in their reports. Two patients with proximal upper extremity STS treated with PBT were included in a small study from the Paul Scherrer Institute [14]. A prospective study from the University of Pennsylvania focused on PBT in previously irradiated fields. Of 23 patients included in the study, the tumor location was in the extremity for 10 patients [21]. Three patients who eventually required amputation after developing recurrence after PBT. Acute wound complications only occurred in 1 patient, but it was not specified if this was a patient with eSTS.

Immobilization is crucial when using PBT for eSTS. Simulation setups used custom fabricated cradles for proximal thigh tumors and thermoplastic mold and mask combinations over the foot and ankle for distal extremity tumors. The reproducibility of the immobilization and positioning was verified with the close collaboration between therapy staff, proton dosimetrist, proton physicist, and physician. An advantage of PBT in this setting is the ability to leave extremities in a more natural position. This is especially relevant for the contralateral extremity because there is less concern regarding low-dose bath compared with photons.

Wound complications are a major cause of morbidity after preoperative photon RT. The seminal National Cancer Institute Canada study demonstrated the use of preoperative radiation before surgical resection of eSTS compared with postoperative radiation was associated with a significantly higher wound complication rate of 35% versus 17% [4]. In addition, patients with lower extremity STS were at particularly high risk for wound complications after preoperative radiation with 43% observed occurrence rate [4]. Compared with photons, protons have a higher entrance dose, potentially leading to more dose at the skin surface and underlying subcutaneous tissues. While historically controlling skin dose with protons has been a concern, pencil beam scanning techniques provide the capacity to modulate skin dose compared with older passive scatter techniques. However, there is still concern that PBT could lead to a higher risk of wound complications compared with photon RT. The results of our study show rates of acute wound complication comparable to historic rates after photon RT suggesting no increased risk after pencil beam scanning PBT.

As the objective of radiation treatment for extremity sarcoma is to encompass the target volume while limiting circumferential dose to radiosensitive structures, such as adjacent bone and skin, more advanced and sophisticated techniques have been investigated [22]. In a phase II trial with 59 patients, O'Sullivan et al [23] demonstrated a wound complication rate of 30.5% with the utilization of IMRT. Image-guided preoperative IMRT to a reduced volume has been implemented to reduce treatment related morbidity for extremity STS [24]. In a retrospective review from Peeken et al [25] imaged-guided helical IMRT (median dose 50 Gy in 25 fractions) was delivered preoperatively on a tomotherapy machine to 41 patients with eSTS. In this patient cohort, the major wound complication rate was 36.8%. The phase II RTOG 0630 trial evaluating the use of image-guided RT to a reduced volume demonstrated a similar acute complication rate of 36.6% (26 of 71) patients [9]. We observed grade 3 acute dermatitis in only 15% of patients and acute wound complication in only 35% of patients.

After surgical resection of a soft tissue sarcoma, soft tissue reconstruction with either free or pedicled flap often is required to achieve limb salvage [26]. These patients are at higher risk for wound complications and tend to be challenging resections involving skin resection or vascular exploration [27]. In our study, 12 of 18 patients (67%) underwent flap reconstruction after excision after preoperative PBT. Considering that patients were selected for PBT because of their large size or challenging anatomic location, these findings are reassuring that PBT does not increase the risk of acute skin toxicity or surgical complications.

A review of a prospective database of 319 patients with eSTS from Memorial Sloan Kettering revealed 5-year local recurrence rates were significantly improved with IMRT 7.6% versus 15.1% [28]. In our study, we report a 1-year locoregional control rate of 100%. Firm conclusions regarding local control will require additional patients and longer follow-up, but this finding suggests that target coverage was not compromised by the improved conformality or planning uncertainties unique to PBT.

Radiation treatment planning for eSTS can often be challenging because of proximity of tumor to bone and the need to spare a strip of lymphatics. Karasek et al [29] demonstrated that increased size of treatment volume was associated with worsening scores for overall function, strength, fibrosis, and skin changes. This was especially true in patients receiving radiation doses higher than 55 Gy [28]. Per Bishop et al [30] a higher risk of fracture was associated with 50 Gy to the entire bone circumference, bone exposure, periosteal stripping during surgery, and use of perioperative chemotherapy. Dickie et al [31] assessed parameters of radiation treatments in 21 patients out of 691 patients who developed bone fracture after treatment of extremity STS. It was demonstrated that the risk of radiation-induced bone fracture was associated with the volume of bone receiving V40 higher than 64%. The fracture incidence was also reduced if the mean dose to bone was < 37 Gy [31]. In patients receiving preoperative PBT in our study, the mean dose to the adjacent bone was 14.1 Gy, potentially reducing the risk of bone fracture compared with other radiation techniques. However, 1 patient who received 66 Gy postoperatively followed by palliative radiation for hip metastasis did develop a pathologic hip fracture requiring hemiarthroplasty. There was a 6-month time span between radiation courses. It is difficult to attribute the cause of fracture given there was metastatic sarcoma in the bone and patient received palliative radiation 6 months after PBT.

The complexity of large, more anatomically challenging STS warrants the use of specialized techniques. In the RTOG 0630 trial using image-guided RT for extremity STS, Wang et al [9] tried to keep soft tissue D50% under 20 Gy and bone D50% < 50 Gy. Gonzalez et al [32] assessed volumetric modulated arc therapy (VMAT) versus sliding window IMRT in postoperative treatment for extremity STS. A significant decrease in D_mean_ in the normal soft tissue was seen in the patients treated with sliding window IMRT versus VMAT (10.4 Gy ± 6.8 Gy vs 14.7 Gy ± 6.5 Gy) [32]. Dosimetry studies focused on PBT versus VMAT for extremity soft tissue sarcoma are also promising. In a study of 10 patients who received 66 Gy postoperatively, Fogliata et al [22] demonstrated that PBT plans had significantly higher dose homogeneity as well as reduced volume receiving medium/low dose levels compared with the VMAT plans the patients received.

Our study represents an early experience and is limited by small patient numbers and short follow-up time. In addition, this small cohort includes patients who received preoperative and postoperative PBT and those undergoing re-irradiation. However, the data suggest that PBT with pencil beam scanning is a promising modality to treat select eSTS cases safely and effectively, with a wound complication rate comparable to historic controls. Patients with larger and anatomically complex lesions may benefit from PBT compared with 3 dimensional conformal photons or IMRT. Long-term follow-up and additional comparison dosimetric data will be crucial in the assessment of tumor control, fibrosis, edema, and quality-of-life outcomes.
